# Probiotic characterization of lactic acid bacteria isolated from swine intestine

**DOI:** 10.14202/vetworld.2017.825-829

**Published:** 2017-07-27

**Authors:** K. Balasingham, C. Valli, L. Radhakrishnan, D. Balasuramanyam

**Affiliations:** 1 Postgraduate Research Institute of Animal Sciences, Tamil Nadu Veterinary and Animal Sciences University, Chennai, Tamil Nadu, India; 2 Institute of Animal Nutrition, Tamil Nadu Veterinary and Animal Sciences University, Chennai, Tamil Nadu, India; 3 Central Feed Technology Unit, Tamil Nadu Veterinary and Animal Sciences University, Chennai, Tamil Nadu, India

**Keywords:** *Lactobacillus acidophilus*, *Lactobacillus plantarum*, probiotic, swine intestine

## Abstract

**Aim:**

A study was conducted with the objective to isolate probiotic microorganisms from swine intestine.

**Materials and Methods:**

In this study 63 isolates (24 caeca, 24 colon mucosal scrapings, and 15 rectal swab samples) were collected from Large White Yorkshire pigs. The isolates were inoculated and grown in de Man Rogosa Sharpe broth at 37°C with 5% CO_2_ for 48 h and subjected to morphological identification. Colonies having Gram-positive rods were selected for further physiological and biochemical identification tests, which were conducted in triplicate in two runs for each of the selected isolates using a standard protocol. Probiotic properties among the identified species were determined through the implementation of several tests related with pH tolerance, bile tolerance, and antimicrobial activity.

**Results:**

Morphological identification revealed that only 23 isolates were Gram-positive rods. Physiological tests performed on these 23 isolates further revealed that four of them did not exhibit any growth, at all conditions studied. The rest 19 isolates were, therefore, selected and subjected to biochemical tests. Six isolates were rejected because they were oxidase and nitrate reduction positive. From the 13 isolates subjected to sugar fermentation tests, speciation of only two isolates could be ascertained, one of the isolates showed characteristics for *Lactobacillus acidophilus* and the other for *Lactobacillus plantarum*. These two isolates were assessed for the strain possessing maximum probiotic property, and it was inferred that both – *L. plantarum* and *L. acidophilus* could tolerate a wide pH range (2-9), a wide bile concentration (0.05-0.3%) and revealed antimicrobial activity toward *Escherichia coli*, and *Enterobacter* spp.

**Conclusion:**

*L. plantarum* and *L. acidophilus* were isolated from swine intestine and were found to have good probiotic properties.

## Introduction

Antibiotics have been widely used for growth parameters in swine [1]. As a result, improved feed efficiency and increased economic returns in swine production were observed. However, pork consumers are increasingly concerned about antibiotic residues in pork [2] and the continuous use of antibiotics could lead to an increased bacterial resistance [3]. Moreover, European Union has banned the use of antibiotics as growth promoters since 2006. Due to these concerns probiotics could play an important role as alternatives to antibiotics in growth promotion.

The contemporary definition of a probiotic is “a microorganism which, when administered in adequate amounts, confers a health benefit on the host” and as living microorganisms, induces no drug resistance or drug residues [4]. Evidence has emerged that probiotics may promote growth, improve feed efficiency, prevent diarrhea and regulate the immune system in pigs. These positive effects are caused by a competitive exclusion of pathogenic bacteria through the colonization of beneficial bacteria in gastrointestinal tract [5]. The prerequisite for a probiotic to favor animals’ performance is the colonization in the gut which is best attained if the organism being administered originates from the gut of same species.

In recent years, numerous probiotic strains have been used in pig production. The application of probiotics provides an alternative strategy to the use of antibiotics [6]. It is well known that not all probiotics are effective in pigs; therefore the selection of the right one is the most time-consuming part of developing a probiotic feed additive that is suitable for pigs [7]. There is, therefore, a need for a host target-specific probiotic strain, screened by appropriate *in vitro* methods that would potentially show enhanced *in vivo* efficacy when administered to livestock as a feed additive [8]. Recently, increased research into the development of probiotics for humans and animals has confirmed that probiotic action may be influenced by the host [9]. The genus *Lactobacillus* could play the role as a probiotic bacterium. This organism is a significant commensal of the normal gut microbiota of mammals and predominant at the early stage of pig gut microflora construction [8].

A study was therefore conducted, to prepare a probiotic preparation specific to swine, by isolating and characterizing the lactic acid bacteria from swine intestine.

## Materials and Methods

### Ethical approval

This study required no ethical approval as the collection of samples were from animals slaughtered for food purpose. All the other experiments were *in vitro*.

### Sample collection

A total of 24 caeca and 24 colon mucosal scrapings were collected immediately after slaughter from 12 male Large White Yorkshire pigs (9-12 months of age) reared in Postgraduate Research Institute in Animal Sciences, Tamil Nadu Veterinary and Animal Sciences University, India. The animals before slaughter were fed swine finisher ration formulated to have 16% crude protein and 3170 Kcal/kg of metabolizable energy. 15 rectal swab samples were collected from 15 live Large White Yorkshire pigs maintained in the same institute. In total, 63 samples *vis-a-vis* isolates were collected.

### Morphological identification

The isolates were inoculated and grown in de Man Rogosa Sharpe (MRS) broth at 37°C with 5% CO_2_ for 48 h and subjected to morphological identification [10].

### Physiological and biochemical tests

Colonies having Gram-positive rods were selected for further physiological (growth at pH 4.5 and pH 9.5, and at NaCl concentration 2% and 6.5%) and biochemical analyses (catalase, nitrate reduction, oxidase, Voges–Proskauer, and production of ammonia from arginine tests, which were carried out in triplicate in two runs for each of the selected isolate using standard protocol as mentioned in manual for the identification of medical bacteria by Cowan and Steel) [11].

### Sugar fermentation tests

Sugar fermentation test [12] was carried out in triplicate in two runs for each of the selected isolates to identify the *Lactobacillus* species. The sugars used in the identification of *Lactobacillus* species were glucose, lactose, arabinose, fructose, esculin, galactose, maltose, mannitol, mannose, melibiose, raffinose, rhamnose, salicin, sorbitol, sucrose, trehalose, and xylose. Each bacterium has its own collection of enzyme that enables it to use diverse carbohydrate; this is often exploited in the identification of bacterial species. Thus, carbohydrate fermentation tests are essential for speciation of bacteria.

### In vitro assays to select Lactobacillus possessing maximum probiotic properties

Probiotic properties among the identified species were determined through pH tolerance test (pH 2, 2.5, 3, 4, 5, 8, and 9), bile tolerance test (0.05%, 0.1%, 0.15%, 0.2%, and 0.3%) test [13], and the measurement of antimicrobial (*Escherichia* *coli* and *Enterobacter* spp.) activity [14]. The above tests were carried out in triplicate for each of the isolates.

### Statistical analysis

The data collected on various parameters were grouped and subjected to statistical analysis by one-way ANOVA using SPSS, version 20.0 for Windows (2011) [15].

## Results

Morphological identification of the isolates revealed that only 23 isolates were Gram-positive rods. Only these isolates were selected for physiological tests. Physiological tests performed on these 23 isolates revealed that four isolates exhibited no growth, irrespective of the examined conditions (pH: 4.5, 9.5; temperature; 15, 45°C; and NaCl: 2, 6.5%). Hence, only 19 isolates were selected and subjected to biochemical tests. During the implementation of the biochemical tests, six isolates were rejected on account that they were oxidase and nitrate reduction positive. From the remaining 13 isolates that were selected and subjected to sugar fermentation tests, speciation of only two isolates could be ascertained. One of the isolates showed characteristics for *Lactobacillus acidophilus* and the other for *Lactobacillus plantarum*.

These two isolates (*L. acidophilus* and *L. plantarum*) were assessed for the strain possessing maximum probiotic property, *viz*., pH tolerance, bile tolerance, and antimicrobial activity.

**Figure-1:**
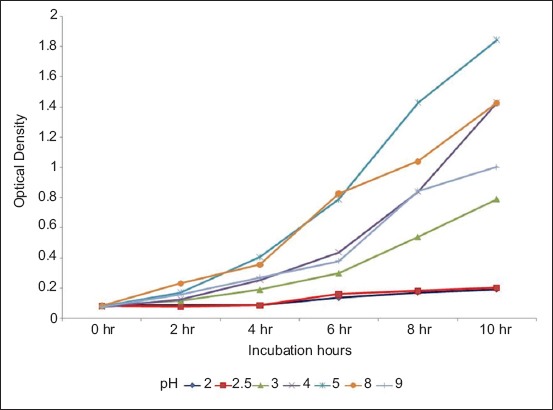
Optical density of de Man Rogosa Sharpe medium containing *Lactobacillus plantarum* at various pH measured at 2 hourly intervals at 650 nm.

**Figure-2:**
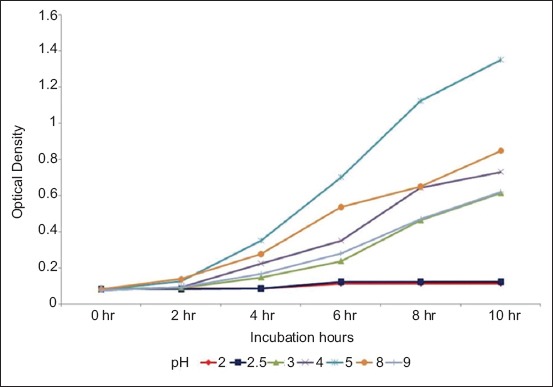
Optical density of de Man Rogosa Sharpe medium containing *Lactobacillus acidophilus* at various pH measured at 2 hourly intervals at 650 nm.

The optical density of MRS medium containing *L. plantarum* and *L. acidophilus* at various pH was measured at 2 h intervals at 650 nm (data are presented in Figures-1 and 2, respectively).

**Figure-3:**
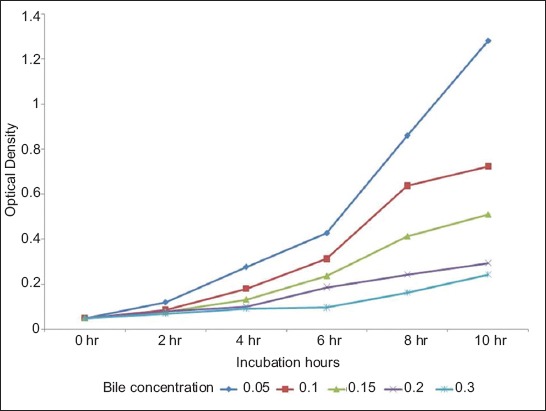
Optical density of de Man Rogosa Sharpe medium containing *Lactobacillus plantarum* at various bile concentrations measured at 2 hourly intervals at 650 nm.

**Figure-4:**
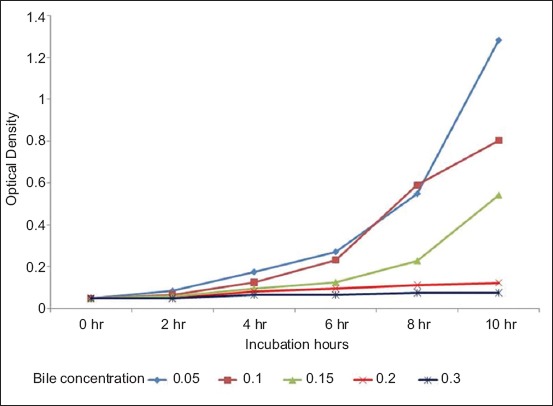
Optical density of de Man Rogosa Sharpe medium containing *Lactobacillus acidophilus* at various bile concentrations measured at 2 hourly intervals at 650 nm.

The optical density of MRS medium containing *L. plantarum* and *Lactobacillus acidophilus* at various bile concentration (%) was also measured at 2 h intervals at 650 nm (data are shown in Figures-3 and 4, respectively).

It was, thus, concluded that both *L. plantarum* and *L. acidophilus* could tolerate a wide pH range (2-9), and a wide bile concentration (0.05-0.3%). *L. plantarum* showed significantly (p<0.05) higher growth compared to *L. acidophilus* at all pH and all bile concentration tested.

The antimicrobial activity of *Lactobacillus* species as demonstrated by the inhibition zone produced (mm) in the agar well diffusion assay is presented in Table-1.

**Table-1:** Antimicrobial activity of *Lactobacillus* spp. as demonstrated by the inhibition zone produced (mm) in the agar well diffusion assay (mean*±SE).

Probiotic organisms	Pathogenic bacteria	

	*Escherichia coli*	*Enterobacter*
*Lactobacillus plantarum*	18.00^a^±0.58	18.33±0.33
*Lactobacillus acidophilus*	20.00^b^±0.59	18.00±0.58

* Mean of six observations.

Means bearing different superscripts within a column differ significantly (p<0.05). SE: Standard error

Both *L. plantarum* and *L. acidophilus* revealed antimicrobial activity toward *E. coli* and *Enterobacter* spp. However, the antimicrobial activity of *L. acidophilus* was significantly (p<0.05) higher compared to *L. plantarum* against *E. coli*.

## Discussion

In this study, the probiotic properties of *L. plantarum* and *L. acidophilus* isolated from pig intestine were shown.

Multidimensional approaches that combine morphological and biochemical data are important for the accurate classification of lactic acid bacteria [16]. Hence, morphological, physiological, and biochemical tests were carried out in this study to isolate *Lactobacilli* from the multiple organisms present in pig intestine. Furthermore, carbohydrate fermentation tests were performed for speciation of isolated bacterial strains.

After the oral administration of probiotic organism, it is subjected to stressing conditions from the host which begins in the stomach with an acid pH between 1.5 and 3 and in the upper intestine and colon where high concentration of bile is encountered. It is, therefore, necessary that an efficient probiotic is capable of growing in an acidic environment and high concentration of bile [17]. As it was shown in this study.

Both *L. plantarum* and *L. acidophilus* could tolerate a wide pH range (3-9), thus fulfilling the criteria that probiotic organisms have to tolerate low pH and also should be capable of growing in a wide range of pH 1-9 [18]. Pyar and Peh [19] had, however, reported that *Lactobacillus* preferred to grow in acidic and neutral environment.

Bile tolerance is another crucial property for probiotic bacteria as it determines the ability of the organism to survive in the small intestine and consequently regulates the capacity of the probiotic to play a functional role [20]. Both *L. plantarum* and *L. acidophilus* could tolerate a wide bile concentration (0.05-0.15%) indicating that both organisms have good probiotic properties. As in this study, it has been demonstrated [21] that when the concentration of bile salts increased there was a decrease in the viability of probiotic organisms. The reason for the reduced growth with increasing level of bile salts could be due to the binding of probiotic organism with bile salts [22].

In this study, both *L. plantarum* and *L. acidophilus* showed antimicrobial activity against *E.coli* and *Enterobacter* spp. The antimicrobial activity could have been due to the effect of organic acids [23] or the production of bacteriocins, which possesses high antimicrobial activity [24]. The production of organic acid and hydrogen peroxide by *Lactobacilli* was reported to inhibit growth of both Gram-positive and Gram-negative bacteria, whereas that of bacteriocin only the growth of Gram-positive bacteria [21].

## Conclusion

*L. plantarum* and *L. acidophilus* were isolated from swine intestine, were found to have satisfactory probiotic properties and could be further exploited as host specific probiotics in swine.

## Authors’ Contributions

The work was carried out by KB as part of MVSc., Research Program, CV, was chairman and LR and DB were the members of advisory committee. All authors read and approved the final manuscript.
